# Early Trajectory of Depressive Symptoms in Alcohol Dependence: Insights From a Four-Week Clinical Decision Window

**DOI:** 10.7759/cureus.110864

**Published:** 2026-06-15

**Authors:** Dhiraj Raja, Alankrita Singh, Harpreet Singh

**Affiliations:** 1 Psychiatry, Heritage Institute of Medical Sciences, Varanasi, IND; 2 Psychiatry, Maharishi Markandeshwar Medical College and Hospital, Solan, IND

**Keywords:** alcohol dependence, alcohol use disorder, audit, depressive symptoms, detoxification, ham-d

## Abstract

Background: Alcohol dependence and depressive symptoms commonly coexist in clinical practice, complicating management and outcomes. While previous studies have largely focused on prevalence and association, the early course of depressive symptoms after detoxification remains unclear despite its clinical importance.

Methods: In this single-group prospective observational study, we included 150 newly diagnosed male patients admitted to a tertiary care hospital in Northern India. We assessed alcohol use severity using the Alcohol Use Disorders Identification Test (AUDIT) and evaluated depressive symptoms using the Hamilton Depression Rating Scale (HAM-D). We provided all patients with standard inpatient detoxification along with routine supportive care. After four weeks of abstinence and supportive inpatient management, we reassessed depressive symptoms and continued naturalistic follow-up for up to 12 weeks after discharge, depending on when patients returned for follow-up.

Results: At admission, 74 patients (49.3%) showed clinically significant depressive symptoms. After four weeks, 29 patients (19.3%) continued to show clinically significant depressive symptoms, while 45 patients (60.8% of those initially symptomatic) showed improvement. HAM-D scores decreased significantly over time (mean reduction 3.92 ± 5.12; p < 0.0001). Alcohol severity showed a positive association with depressive symptoms at both baseline and four weeks.

Conclusion: These findings suggest that while many patients improve during early abstinence and supportive inpatient management, a substantial proportion continue to experience depressive symptoms. The early postdetoxification period may provide a clinically useful timeframe for reassessment and further management planning.

## Introduction

In day-to-day clinical practice, we often see alcohol dependence and depressive symptoms together. Managing these patients is rarely straightforward. Most patients with alcohol dependence also present with depressive symptoms such as low mood, poor sleep, loss of interest in routine activities, and occasionally suicidal thoughts, which together make diagnosis and treatment more challenging. We diagnose alcohol dependence using standard criteria such as the International Classification of Diseases 11th Revision [1]. Alcohol dependence continues to contribute significantly to global morbidity, psychosocial burden, and healthcare challenges, making it a major public health concern worldwide [2].

India shows a similar pattern, with increasing alcohol use contributing to a growing clinical and public health burden across different population groups, including younger individuals [3]. National surveys have also shown that substance use is widespread across the country [4]. At the same time, depressive symptoms are commonly seen in people with alcohol dependence [5]. In routine clinical practice, it is uncommon to see alcohol dependence without associated emotional or psychological symptoms.

The relationship between alcohol use and depressive symptoms is complex and bidirectional. Some individuals with depression later increase alcohol use, while others begin drinking to cope with stress or emotional distress [6,7]. Studies have reported depressive symptoms in nearly 30%-60% of individuals with alcohol dependence, depending on the population studied [5,8]. Although alcohol may provide temporary relief initially, long-term use often worsens mood, sleep, and overall psychological well-being. This creates a bidirectional cycle in which depressive symptoms increase alcohol use, while continued alcohol use further aggravates depressive symptoms [6]. In some patients, these symptoms do not improve completely even after stopping alcohol [7]. Reviews have also shown that mood symptoms are very common in people with substance use disorders [8].

Indian studies show a similar picture, but most of them are cross-sectional [9]. They show how common depressive symptoms are, but they do not clearly explain what happens after treatment starts. This creates a practical clinical challenge because clinicians often remain uncertain whether to continue observation or begin treatment early. People often use alcohol as a coping mechanism, and stressful situations are closely linked to both alcohol use and depressive symptoms [10,11].

Over time, alcohol use and depressive symptoms continue to influence each other. Long-term studies show that both conditions often develop together and continue to affect each other [12]. As a result, clinicians usually manage both conditions together [13]. Some studies also suggest that both conditions may gradually develop side by side [14], and recent data also show a high burden of psychiatric comorbidity in alcohol-associated conditions [15].

Not all patients follow the same pattern. Some improve after stopping alcohol, while others continue to have depressive symptoms even after detoxification [16]. This difference is clinically important during decision-making. At the same time, access to proper mental health care remains limited in many settings [17]. Simple psychosocial measures such as counseling and group therapy can help, but they are not always available [18]. Indian literature also shows that although the problem is increasing, limited research has examined how these symptoms change over time [19].

Persistent depressive symptoms are clinically important because they increase the risk of relapse [20]. Alcohol also affects brain systems involved in mood regulation and stress response, which may contribute to persistent depressive symptoms in some individuals [21,22]. We assessed alcohol use severity and depressive symptoms using standard clinical tools such as the Alcohol Use Disorders Identification Test (AUDIT) and Hamilton Depression Rating Scale (HAM-D) [23,24]. Many patients also use alcohol to manage their mood symptoms, which further complicates the clinical picture [25]. Overall, clinicians often need a simple and stepwise approach to manage these patients effectively [26].

Despite all this, most studies focus mainly on the prevalence of depressive symptoms and do not clearly describe what happens during the early weeks after stopping alcohol. This period is clinically important because clinicians often need to decide whether to continue observation or begin treatment early.

This is where the present study becomes relevant. Instead of focusing only on prevalence, we examined how depressive symptoms changed after detoxification. Some patients improved during early abstinence and supportive inpatient management, whereas others continued to experience persistent symptoms requiring further clinical attention.

By focusing on this early phase, we aimed to provide a more practical understanding of early changes in depressive symptoms in alcohol dependence and their relevance to clinical reassessment during abstinence.

Keeping this in mind, we conducted this prospective observational study to assess the prevalence of depressive symptoms in newly diagnosed alcohol-dependent patients at admission and to evaluate early changes in depressive symptoms after structured detoxification and supportive inpatient management over four weeks. We also aimed to observe the proportion of patients who continued to experience persistent depressive symptoms during early abstinence.

## Materials and methods

Study design and setting

We conducted this single-group prospective observational study at a tertiary care hospital in North India. We assessed patients at admission and followed them during their hospital stay to evaluate early changes in depressive symptoms after detoxification. The primary study endpoint was the Day 28 reassessment following abstinence and supportive inpatient management. We did not create separate case-control or exposed/nonexposed groups because we followed a single cohort of alcohol-dependent patients longitudinally over time.

After discharge, follow-up remained naturalistic and depended on when patients returned on their own in routine clinical practice. Because patients with persistent symptoms or relapse were more likely to return for follow-up, we interpreted observations beyond the primary four-week endpoint cautiously and considered them exploratory.

The Institutional Ethics Committee approved the study, and we obtained written informed consent from all patients before their inclusion.

Participants

During the study period, we screened 180 patients with alcohol-related problems. Of these, we included 150 male patients who met the study criteria. We included only male patients because very few female patients with alcohol use problems presented to the hospital during the study period.

Inclusion and exclusion criteria

The inclusion and exclusion criteria are shown in Table 1.

**Table 1:** Criteria used for inclusion and exclusion of participants in the study ICD-11: International Classification of Diseases 11th Revision; AUDIT: Alcohol Use Disorders Identification Test

Inclusion criteria	Exclusion criteria
Alcohol dependence as per ICD-11	Serious medical illness
Age ≥18 years	Neurological illness
AUDIT score ≥13	Major psychiatric illness (other than depression)
	Already on antidepressants

Assessment tools

Alcohol Use Disorders Identification Test

We used AUDIT to assess the severity of alcohol use. It is a widely used WHO-developed tool that evaluates drinking patterns and related problems [23].

Hamilton Depression Rating Scale

We used HAM-D primarily to assess the severity of depressive symptoms rather than as a standalone diagnostic tool for major depressive disorder [24]. Treating psychiatrists also performed routine clinical psychiatric evaluations at admission as part of the standard inpatient assessment. We used AUDIT and HAM-D as widely accepted clinical tools for academic and noncommercial research purposes.

Study procedure

Baseline (Day 1)

At admission, we conducted a clinical psychiatric assessment of the patients. We assessed alcohol use severity using AUDIT and evaluated depressive symptoms using HAM-D.

Treatment and Detoxification

All patients received standard detoxification treatment. We used medications such as benzodiazepines to control withdrawal symptoms. We administered vitamin B complex and thiamine to prevent complications and monitored patients regularly throughout their hospital stay.

Along with standard detoxification treatment, we provided routine supportive care, including group therapy sessions, Alcoholics Anonymous (AA) meetings, and supportive practices such as yoga and relaxation. These interventions formed part of routine inpatient care, although we did not formally quantify participation intensity or individual engagement.

We did not start antidepressants during the first 28 days because we wanted to observe early changes in depressive symptoms during abstinence and supportive inpatient management, as suggested in earlier studies [6,16].

Follow-Up (Day 28)

After about four weeks, we reassessed patients using HAM-D to evaluate changes in depressive symptoms. We chose this time point because it represents an early and clinically important phase after stopping alcohol [21].

Outcome measures

We assessed three main outcomes: the number of patients with clinically significant depressive symptoms at baseline, the change in HAM-D scores after four weeks, and the number of patients with persistent depressive symptoms after detoxification.

Statistical analysis

We analyzed the data using descriptive and exploratory statistical methods. We expressed values as means and percentages wherever appropriate. We compared HAM-D scores at baseline and after four weeks. We assessed the relationship between alcohol severity and depressive symptoms using correlation analysis. We used the McNemar test to assess changes in depressive symptom status over time [27]. We considered a p value of less than 0.05 as statistically significant. We did not analyze some factors, such as duration of alcohol use or nicotine use, in detail in this study.

## Results

Sample characteristics

We included 150 participants in the study. All participants were male and met the criteria for Alcohol Dependence Syndrome.

Baseline prevalence of depression

At baseline, 74 patients (49.3%) showed clinically significant depressive symptoms (HAM-D > 9), while 76 patients (50.7%) did not show significant depressive symptoms.

Change in depression after 28 days

After four weeks of abstinence and supportive care, 29 patients (19.3%) continued to show clinically significant depressive symptoms. Among those who showed depressive symptoms at baseline, 45 patients (60.8%) no longer showed clinically significant depressive symptoms after four weeks. The remaining 76 patients who did not show significant depressive symptoms at baseline continued to remain free of significant depressive symptoms.

**Figure 1:**
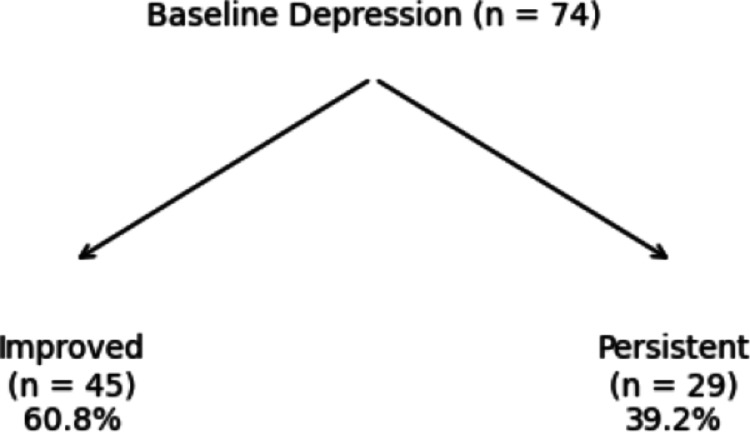
Early clinical trajectory of depressive symptoms after detoxification The distribution of patients into improved and persistent groups after four weeks is shown

Table 2 shows the change in depressive status from baseline to Day 28. These findings reveal two early clinical trajectories: improvement in the majority of patients and symptom persistence in a smaller group. Figure 1 shows this early clinical trajectory.

**Table 2:** Change in depressive status from baseline to Day 28 (n = 150) Values are presented as number (%)

Baseline status	Depression at Day 28	No depression at Day 28	Total
Depression (n = 74)	29 (39.2%)	45 (60.8%)	74
No depression (n = 76)	0 (0%)	76 (100%)	76

Reduction in HAM-D scores

**Figure 2:**
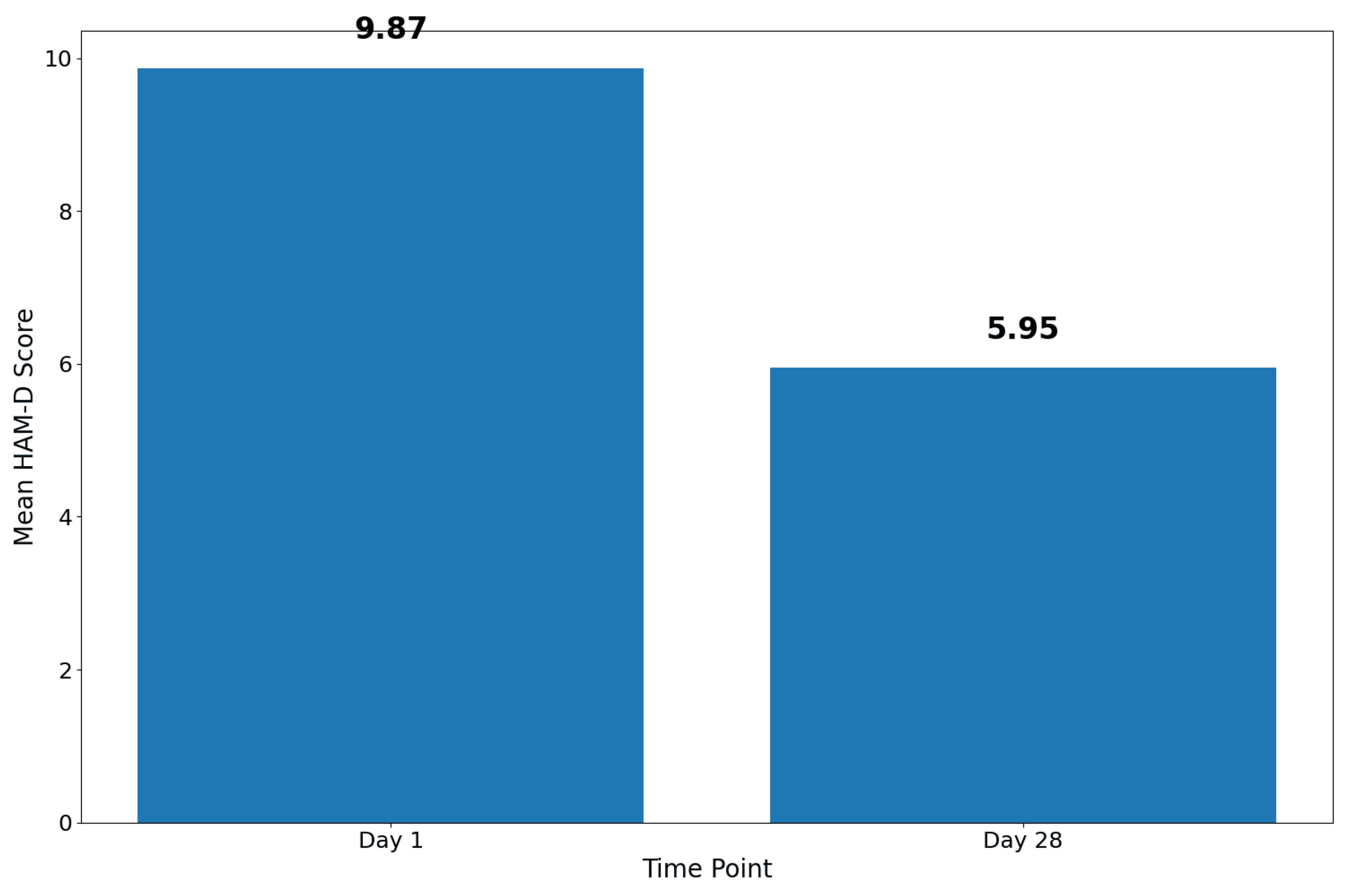
Change in HAM-D scores from Days 1 to 28 Graph showing reduction in HAM-D scores from baseline to four weeks HAM-D: Hamilton Depression Rating Scale

**Figure 3:**
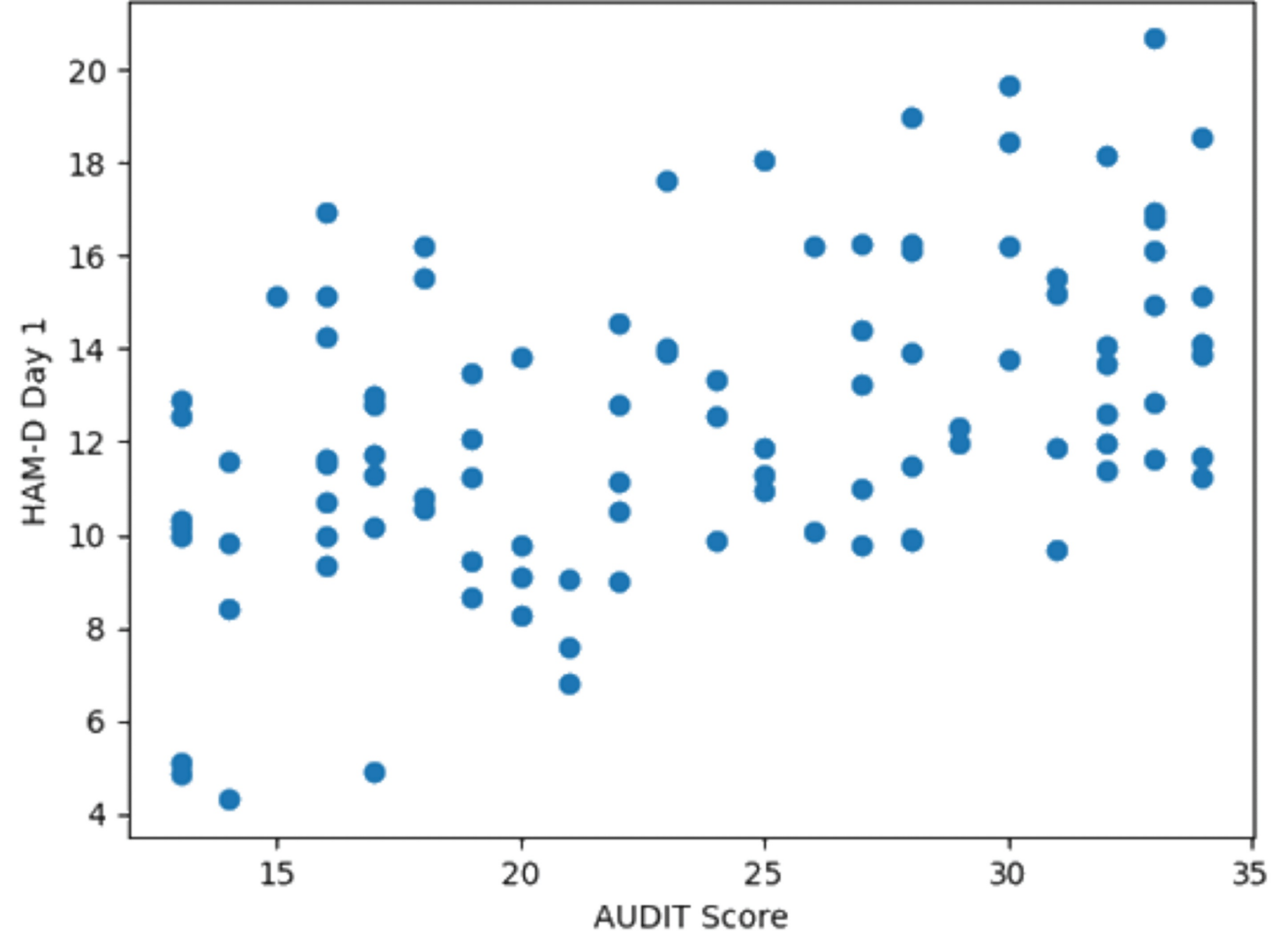
Association between AUDIT score and HAM-D score at Day 1 (baseline) AUDIT: Alcohol Use Disorders Identification Test; HAM-D: Hamilton Depression Rating Scale

**Figure 4:**
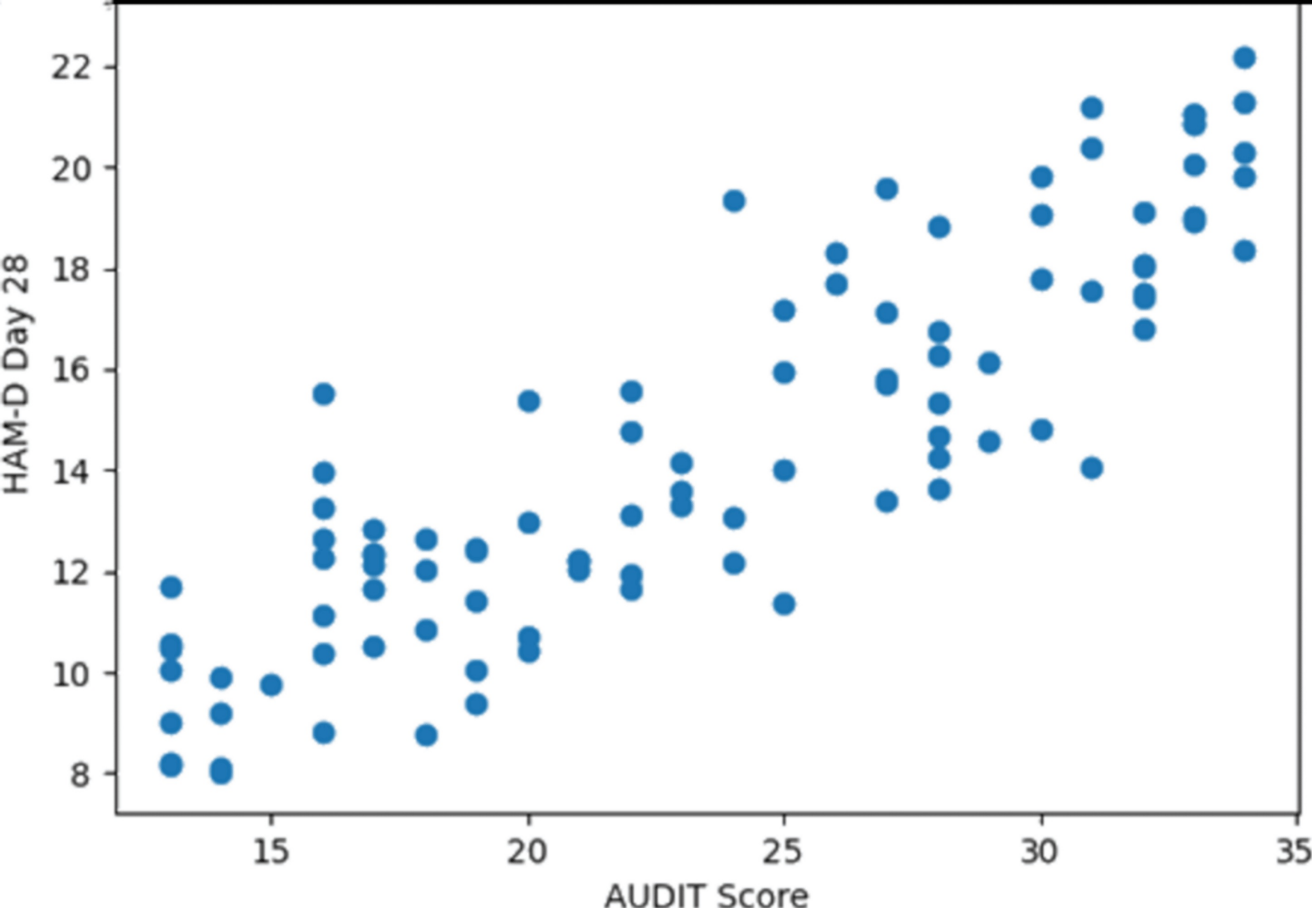
Association between AUDIT score and HAM-D score at Day 28 AUDIT: Alcohol Use Disorders Identification Test; HAM-D: Hamilton Depression Rating Scale

We observed a positive association between AUDIT scores and HAM-D scores at both baseline and Day 28 (p < 0.0001) (Figure 2). Figure 3 shows the baseline association, and Figure 4 shows the association at Day 28.

Correlation between alcohol severity and depression

We found a positive correlation between AUDIT scores and HAM-D scores at both baseline and Day 28 (p < 0.0001). Figure 3 shows the baseline correlation, and Figure 4 shows the correlation at Day 28.

Nonsignificant association between alcohol severity and change in depressive symptoms

We did not observe a significant association between alcohol use severity and the magnitude of change in HAM-D scores over time (p = 0.681). This finding suggests that higher AUDIT scores were not consistently associated with greater improvement or worsening in depressive symptoms during the early observation period.

## Discussion

In this study, almost half of the patients had depressive symptoms at the time of presentation. After stopping alcohol and receiving structured care, the number of patients with depressive symptoms reduced, although not completely. Many patients improved, but about one-fifth continued to experience depressive symptoms. These findings suggest that depressive symptoms in alcohol dependence do not follow a uniform clinical course.

Most earlier studies, both from India and other countries, have mainly focused on prevalence or long-term outcomes. They consistently report that a significant proportion of patients with alcohol dependence have depressive symptoms, often in the range of 30%-60% [5,8], and Indian data also reflect a similar pattern [3,4]. However, most of these studies provide only a cross-sectional view and do not clearly describe what happens after patients stop drinking alcohol.

In this study, we focused on the early phase after detoxification. We found that patients tended to follow two broad clinical patterns. Some patients showed early improvement during abstinence and supportive inpatient management, whereas others continued to experience depressive symptoms. These observations broadly suggest two early clinical patterns: one group shows improvement during early abstinence and supportive inpatient management, whereas the other continues to experience persistent symptoms. This distinction has practical value in clinical decision-making.

At the same time, early depressive symptoms may overlap with alcohol withdrawal-related mood changes, so clinicians should interpret these findings carefully during the initial phase.

The difference in early response suggests that depressive symptoms in alcohol dependence do not follow a uniform clinical pattern. In some patients, depressive symptoms may be closely related to alcohol use. They may improve during abstinence and supportive inpatient management, whereas in others, these symptoms may reflect a more persistent or independent clinical condition [7,12,16].

Most studies acknowledge that alcohol dependence and depression commonly occur together, but they do not clearly explain how clinicians can use this information in early clinical practice. Our findings suggest that careful observation during the early abstinence period itself can provide useful clinical clues.

We also observed that patients with more severe alcohol use were more likely to have persistent depressive symptoms. This observation is consistent with existing evidence. Long-term alcohol use affects brain systems involved in mood, stress, and reward, and these changes may not reverse immediately after stopping alcohol [15,22,26]. This may explain why some patients continue to experience symptoms even after detoxification.

Psychological factors also play an important role. Many patients use alcohol to cope with stress, loneliness, or emotional distress. Although alcohol may provide short-term relief, it often worsens the underlying problem over time. This creates a cycle in which depressive symptoms increase alcohol use, while continued alcohol use further aggravates depressive symptoms [7,12].

An important finding in this study is that many patients improved with relatively simple interventions, including detoxification, supportive care, group therapy, and peer support such as AA. This finding is particularly relevant in low-resource settings, where access to specialized care may remain limited. It shows that even structured basic care can lead to meaningful improvement.

At the same time, the group that did not improve remains clinically important. Clinicians may overlook these patients if they assume that all depressive symptoms will resolve after stopping alcohol. Our findings suggest that this assumption does not apply to all patients.

This study differs from many previous studies by connecting clinical observation with practical decision-making. Instead of reporting only prevalence, it examines how patients behave in the early phase after detoxification. This has direct clinical relevance. Rather than initiating antidepressants immediately in all patients, clinicians can observe patients for a short period and then decide on treatment based on clinical response.

These findings support a stepwise clinical approach involving initial detoxification, a short observation period, reassessment after a few weeks, and targeted treatment for patients who continue to experience persistent symptoms. This approach may also help clinicians identify patients who require further clinical evaluation for persistent depressive symptoms in routine clinical practice.

Overall, this study reflects real-world clinical patterns rather than the controlled experimental conditions that clinicians often find difficult to replicate in routine settings.

This approach can help clinicians avoid both overtreatment and undertreatment. Patients who improve early may not require additional medication, whereas clinicians can more appropriately identify and manage patients with persistent symptoms. This approach aligns with routine clinical practice, although many previous studies have not clearly emphasized its practical value.

Another important observation is that most Indian studies remain cross-sectional and do not capture longitudinal changes. Even globally, although researchers have well established the association between alcohol use and depressive symptoms, they have not widely emphasized the practical relevance of early symptom changes in clinical decision-making. This study adds a small but clinically meaningful perspective in that direction.

Finally, persistent depressive symptoms have important clinical implications. Patients who continue to experience depressive symptoms are more likely to relapse, show poor adherence, and have worse long-term outcomes [20,26]. Identifying these patients early can help clinicians improve treatment planning and overall outcomes.

Strengths and novel contributions

This study adds value because it goes beyond prevalence and examines what happens after patients stop alcohol [5,16]. One important observation is that patients do not follow a single clinical pattern; some improve early, whereas others continue to experience symptoms. This supports the idea that depressive symptoms in alcohol dependence may follow different clinical pathways [7,16].

Another important contribution is the focus on the early post-detoxification period. The first few weeks, especially around the fourth week, may provide a practical period for clinical reassessment and treatment planning. During this period, clinicians may better understand whether depressive symptoms are more likely related to alcohol use or reflect a more persistent clinical condition [26].

Because we conducted the study in an inpatient setting and maintained abstinence during admission, ongoing alcohol use was less likely to influence the findings. From a practical perspective, identifying patients with persistent depressive symptoms remains important because these patients face a higher risk of relapse and poorer outcomes [20,26].

Limitations

We conducted this study in a single tertiary-care inpatient setting and included only male patients, which may limit the generalizability of the findings to other populations. We primarily focused on the early phase after detoxification and did not assess longer term outcomes in detail [20,26]. Because we assessed depressive symptoms during early abstinence using HAM-D, some overlap between depressive symptoms and alcohol withdrawal-related symptoms may have occurred. We did not perform structured diagnostic interviews specifically to differentiate alcohol-related depressive symptoms from independent depressive disorders.

We also did not formally quantify the intensity of participation or engagement in psychosocial interventions such as group therapy, AA meetings, yoga, or relaxation practices. Therefore, we cannot attribute improvement in depressive symptoms solely to abstinence. In addition, we did not analyze several potentially relevant confounding factors, including nicotine dependence, psychosocial stressors, family history, or previous depressive episodes, in sufficient detail for adjusted multivariable analysis.

We did not initiate antidepressants during the early phase because we intended to observe the natural early course of depressive symptoms during abstinence and supportive inpatient management. Despite these limitations, the study reflects real-world clinical practice and provides a practical understanding of early changes in depressive symptoms during abstinence and supportive inpatient management.

Future perspectives

Future studies should follow patients over a longer duration to better understand outcomes beyond the early post-detoxification phase. Researchers should also identify early predictors of persistent depressive symptoms, including severity of alcohol use, withdrawal severity, psychosocial stressors, nicotine dependence, and previous depressive episodes. Further research should clarify the optimal timing for initiating antidepressant treatment and determine how clinicians can best manage coexisting alcohol dependence and depressive symptoms, particularly in low-resource settings.

Future research should also use structured diagnostic interviews and standardized psychosocial intervention protocols to differentiate alcohol-related depressive symptoms from more persistent depressive disorders more clearly. In the Indian context, improving awareness, early identification, and access to mental health services may further improve long-term outcomes.

## Conclusions

Depressive symptoms commonly occur in patients with alcohol dependence, but patients do not show a uniform pattern of improvement during the early post-detoxification period. While many patients show improvement during abstinence and supportive inpatient management, a substantial proportion continue to experience persistent depressive symptoms.

The early weeks after detoxification may provide a clinically useful period for reassessment and follow-up. A practical stepwise approach involving detoxification, short-term observation, reassessment, and targeted management may help clinicians identify patients who continue to experience depressive symptoms and may benefit from further clinical evaluation and treatment.
